# Implementation and Core Components of a Multimodal Program including Exercise and Nutrition in Prevention and Treatment of Frailty in Community-Dwelling Older Adults: A Narrative Review

**DOI:** 10.3390/nu15194100

**Published:** 2023-09-22

**Authors:** Leo Delaire, Aymeric Courtay, Joannès Humblot, Mylène Aubertin-Leheudre, France Mourey, Antoine Noël Racine, Thomas Gilbert, Zeinabou Niasse-Sy, Marc Bonnefoy

**Affiliations:** 1 Service de Médecine du Vieillissement, Hôpital Lyon Sud, Hospices Civils de Lyon, 69495 Pierre-Bénite, France; aymeric.courtay@chu-lyon.fr (A.C.); joannes.humblot@chu-lyon.fr (J.H.); thomas.gilbert@chu-lyon.fr (T.G.); zeinabou.niasse-sy@chu-lyon.fr (Z.N.-S.); marc.bonnefoy@chu-lyon.fr (M.B.); 2 Programme «Bien sur ses Jambes», Hôpital Lyon Sud, Hospices Civils de Lyon, 69495 Pierre-Bénite, France; 3 Centre de Recherche de L’Institut Universitaire de Gériatrie de Montréal (CRIUGM), Montréal, QC H3W 1W5, Canada; aubertin-leheudre.mylene@uqam.ca; 4 Groupe de Recherche en Activité Physique Adaptée, Département des Sciences de l’Activité Physique, Université du Québec à Montréal (UQÀM), Montréal, QC H2L 2C4, Canada; 5 Laboratoire CAPS (Cognition, Action, et Plasticité Sensorimotrice), Inserm U1093, UFR STAPS, Université de Bourgogne, Campus Universitaire, BP 27877, 21078 Dijon, France; france.mourey@u-bourgogne.fr; 6 Université Côte D’Azur, LAMHESS, 06205 Nice, France; antoine.noel-racine@univ-cotedazur.fr; 7 RESHAPE Research on Healthcare Professionals and Performance, Inserm U1290, Université Claude Bernard Lyon 1, 69008 Lyon, France; 8 Inserm U1060-CarMeN, Université Claude Bernard Lyon 1, 69100 Villeurbanne, France

**Keywords:** frailty, prevention, exercise, nutrition, program

## Abstract

Increasing disability-free life expectancy is a crucial issue to optimize active ageing and to reduce the burden of evitable medical costs. One of the main challenges is to develop pragmatic and personalized prevention strategies in order to prevent frailty, counteract adverse outcomes such as falls and mobility disability, and to improve quality of life. Strong evidence reports the effectiveness of exercise interventions to improve various physical parameters and muscle function that are cornerstones of frailty. Other findings also suggest that the interactions between nutrition and physical exercise with or without health behavior promotion prevent the development of frailty. Multimodal programs, including structured exercise, adequate dietary intervention and health behavior promotion, appear increasingly consensual. However, in order for implementation in real-life settings, some pitfalls need to be addressed. In this perspective, structuring and tailoring feasible, acceptable and sustainable interventions to optimize exercise training responses are essential conditions to warrant short, medium and long-term individual benefits. The different components of exercise programs appear to be fairly consensual and effective. However, specific composition of the programs proposed (frequency, intensity, type, time, volume and progressiveness) have to be tailored to individual characteristics and objectives in order to improve exercise responses. The intervention approaches, behavioral strategies and indications for these programs also need to be refined and framed. The main objective of this work is to guide the actions of healthcare professionals and enable them to widely and effectively implement multimodal programs including exercise, nutrition and behavioral strategies in real-life settings.

## 1. Introduction

Frailty is a complex but recognizable geriatric syndrome defined as a clinical state in which the ability of older adults to cope with everyday or acute stressors is compromised by an increased vulnerability brought about by age-associated declines in physiological reserve and function across multiple organ systems [1]. Frailty was originally described as physical and cumulative deficits, characterized by the Frailty Phenotype (FP) as unintentional weight loss, self-reported exhaustion, physical inactivity, slowness and weakness [2]. The etiology of frailty remains uncertain but evidence suggests an interaction with genetic factors, nutrition, social determinants, cognitive decline and sedentary behaviors [3]. Thus, pathogenesis of frailty is multidimensional and requires an approach that encompasses medical, environmental and social domains to also monitor intrinsic capacity as proposed by the World Health Organization (WHO) (i.e., locomotion, vitality, cognition, psychological, sensorial) [4,5]. Pre-frailty, an intermediate clinical state between robustness and frailty, is also a widespread syndrome in older adults [6,7,8]. In a systematic review and meta-analysis, the incidence rate was approximately 151 and 43 new cases per 1000 person-years for pre-frailty and frailty, respectively [7]. Although being more prevalent in hospitalized and institutionalized older adults, community-dwelling older adults are also prone to develop frailty [7,8]. While frailty accelerates the decline of functional capacity with aging, it remains a preventable and reversible condition [4,9].

The main mediator that counteracts frailty occurrence and its progression is physical activity (PA) [10,11,12]. A sedentary lifestyle worsens the functional trajectory of aging and is associated with frailty, sarcopenia and mobility disability [11,13,14]. An observational study, including 1529 community-dwelling older adults, showed that low PA, defined by the FP index, was the main predictor of frailty [15]. Thus, promoting active aging through regular PA is of the utmost relevance to increase years lived without mobility disability, improve quality of life and reduce evitable medical costs, in particular in high-risk frail individuals (>3 criteria in the FB) [16,17,18,19,20]. In this regard, strategies resolutely focusing on PA are essential to prevent the onset of frailty and its detrimental effects [4,21].

The guidance of healthcare professionals (HCP) through primary and secondary preventive strategies developed towards pragmatic, personalized, participative and cost-effective approaches is essential [20,22,23,24,25,26]. Strong evidence supports the effectiveness of structured exercise interventions to improve various physical parameters and muscle function that are cornerstones of frailty and sarcopenia [27,28,29]. Evidence also suggests that adding nutrition to exercise programs reinforces its effectiveness [29,30,31]. Moreover, health literacy promotion and behavioral strategies appear also to be important interventions to foster older adults’ involvement in health-related behaviors, such as regular PA [32,33,34,35]. Thus, although multicomponent exercise programs are generally recognized as a reference strategy to prevent frailty, practical implementation on a wide scale remains challenging due to intra and interpersonal factors, human resources, environment and involvement of stakeholders [23,34]. In this perspective, implementing structured and tailored exercise programs must be in line with feasibility, acceptability, fidelity and sustainability to optimize exercise responses and to ensure long-term individual uptake [20,23,36,37]. These conditions need to be completed to warrant individual benefits in the short- and, mostly, in the medium- and long-term [14,17,38,39,40,41].

It Is generally admitted that exercise programs for older adults with frailty should include resistance (strength and power), endurance, balance and functional training; all of these types can be combined into multicomponent exercise training [21,27,42,43]. In a complementary way, other type of exercise might include Nordic walk, tai-chi or yoga [44,45,46]. The different components of exercise programs appear to be fairly consensual and effective. However, the specific composition of the programs proposed (frequency, intensity, type, time, volume and progressiveness) has to be tailored to individual characteristics and objectives in order to improve exercise responses. The intervention approaches, behavioral strategies and indications for these programs need also to be refined and framed [47]. The main objective of this work is to guide the actions of HCP and enable them to widely and effectively implement multimodal programs including exercise, nutrition and behavioral strategies in real-life settings. In this narrative review, we have focused on the following points: exercise components and intervention settings description for the prevention and treatment of frailty. In addition, we briefly discussed behavioral strategies, nutrition and the synergistic approach between exercise and nutritional supplementation.

## 2. Method

We conducted a narrative review to update the available literature on the field of the prevention and treatment of frailty based on exercise interventions, nutrition and/or both combined. We searched for the study selection on PubMed, Medline and Google Scholar. We selected published articles in the last twenty years (2003–2023). A particular interest was on recent systematic reviews and meta-analyses (between 2018 and 2023). Older articles (prior to 2003) were kept in selection in some cases when considered relevant for the narrative review (i.e., princeps studies). A search strategy including the following terms was used: [frail older adults]; [frailty]; [exercise training]; [exercise interventions]; [nutrition exercise]; [nutritional supplementation]. Exclusion terms were used in some cases, such as NOT [healthy older adults]. We performed backward citation tracking to increase the number of selected publications and evidence. Publications selected were mainly related to community-dwelling older adults living with frailty, preferably defined by the FP. Publication disciplines included sports science, especially adapted physical activity (exercise training, exercise physiology and kinesiology), gerontology, public health, behavioral sciences and human nutrition. We placed a strong emphasis on publications that provided evidence to address gaps in practice and knowledge. Tables were used to gather studies and to note their main characteristics (population, methods, main objective, results, conclusions, perspectives), as recommended by others [48]. We used the Scale for the Assessment of Narrative Review Articles (SANRA) to prepare a qualitative selection of studies supporting the narrative review [49]. We included a total of 344 studies in the review. Finally, we made a synoptic table (Table 1) proposing the main characteristics of a multimodal program composition, including exercise, nutrition and behavioral strategy, in order to prevent and treat older adults with frailty. We summarize each sections at the end by key points, gathered in another synoptic table (Table 2).

## 3. Screening Strategies

### 3.1. Screening Tools

Determining an efficient screening strategy is challenging for HCP as multiple and distinctive approaches exist to describe frailty [83]. Most of the instruments used for screening purposes display a relative poor agreement, a high heterogeneity for detecting frailty and require additional resources [84,85]. The screening of older adults at risk of frailty must be based on a performant, feasible and predictive indicator designed for the intended setting and population in order to implement a targeted and personalized management of frailty [4,84,86]. In primary care, screening instruments must be rapid, easy to uptake and applicable [84]. In this regard, the FRAIL scale, The Clinical Frailty Scale and the Gérontopole Frailty Screening Tool (GFST) are appropriate considering their time-effectiveness and feasibility [87]. In the same study, frailty prevalence was higher using the GFST (32.2%), the SHARE Frailty Instrument (30.5%) and the FP (16.2%) [87]. However, a recent study concluded that in primary care, none of these instruments were predictable of adverse outcomes, displaying a poor sensibility and specificity [88]. Alternatively, the electronic frailty index, an automated method developed from routine primary care data, represents a promising solution with robust predictive values for several frailty outcomes (i.e., hospitalization, institutionalization and mortality) [89].

### 3.2. Physical Performances

The use of physical performances to screen physical frailty may be relevant as they are strongly related to functional decline, risk of adverse outcomes and overlap several approaches of frailty [90,91,92,93]. A systematic review and meta-analysis showed that the strongest correlation obtained for physical fitness components with frailty was usual gait speed, regardless of the method used [93]. This last point is consistent with the age-related change in function which emphasizes the importance of the rate of decline in gait speed to predict mobility disability [91]. Moreover, gait speed performances are strongly associated with functional independence and quality of life in older adults [94,95,96]. Recently, a longitudinal analysis on 3070 older adults showed that low gait speed, as defined by the FP (≤0.65 m.s and ≤0.75 m.s regarding gender and height), was the only discriminator of incident disability regarding basal and instrumental activities of daily living [97]. Additionally, it was demonstrated that six spatio-temporal gait parameters were associated with frailty and that stride variability under fast conditions was the most prominent variable [98]. Yet, it remains essential to determine clinical changes in usual gait speed relative to the onset of self-reported mobility disability [99]. While gait speed tends to be a determinant factor in the apparition of frailty, it is also noteworthy to mention the five repetitions Sit-To-Stand (5-STS) performance for screening purposes as it is associated with global muscle weakness and strongly predicts falls incidence [100,101]. Furthermore, 5-STS represents the gold standard for reduced functional capacity in the Integrated Care for Older PEople (ICOPE) program [5].

### 3.3. Physical Activity

Supported evidence shows that poor energy expenditure leads to an increased risk of developing frailty and that sedentary behaviors increase the risk of mortality in older adults with frailty [3,11,102]. However, PA (PA) remains rarely embedded among the variety of frailty approaches. Physical inactivity (i.e., <150 min of moderate PA per week or <90 min of vigorous PA per week) and/or sedentary behaviors (i.e., activities close to resting energy expenditure <3 Metabolic Equivalent of Tasks or METs) should also be considered as warning signs when screening physical frailty as it was associated with higher frailty prevalence, incident disability in autonomy for daily living and mortality [11,97,103]. HCP could also use digital technologies to more objectively address PA (i.e., intensity and volume) and, thus, improve screening measures. Recent studies showed that higher levels of PA (i.e., less time in sedentary activities) measured by wearable accelerometers was associated with less odds of being frail [104,105,106].

### 3.4. Self-Screening

With the complexity of integrating screening procedures of frailty into routine care (time-consuming, lack of resources, etc.), self-screening tools represent an important opportunity to move forward in this field. It may also be of interest to avoid unnecessary Comprehensive Geriatric Assessments (CGA) given the scarcity of resources in particular in low-income countries and rural communities [107]. Interestingly, self-reported frailty and test-based measures of frailty (e.g., gait speed, grip strength, etc.) highlight frailty similarly in community-dwelling older adults [108]. Furthermore, older adults self-screened at risk of frailty might be able to empower themselves in the management of their health [107]. Remote self-screening by smartphone applications may be meaningful to manage frailty screening on a larger scale. For instance, the ICOPE program encourages older adults to assess their own intrinsic capacity every 4–6 months by using a website or a dedicated application [5]. However, self-screening based on mobile devices or websites requires cultural adoption, sufficient literacy and tailored configuration in order to improve its feasibility [109]. In older adults with mild cognitive impairment or dementia, wearable accelerometers measuring PA might be of interest to detect frailty, although lack of standardization methods, protocols and metrics limit the level of evidence [110,111].

**Key points**

- Screening strategy has to be based on performant and feasible tools designed for the intended setting.
- Physical performances could be appropriate surrogates of frailty indexes and can be easily assessed.
- PA is a predominant risk factor of frailty and must be a primary variable to assess.
- Self-screening using new technologies represents a great opportunity to move forward in this field and can be used with confidence.

## 4. Assessments Methodologies

While the majority of screening tools display a sufficient sensibility to rule out frailty in clinical practice, their specificity remains low in community-dwelling settings [112]. At-risk older adults screened positively should therefore be assessed more thoroughly within a complete evaluation as part of a CGA [84]. Prior to an exercise intervention, a CGA may help to reduce frailty [52]. As such, investigations should notably assess an in-depth evaluation of the components of frailty, physical and functional capacities, nutritional status, level of PA and must also dismiss potential contraindications to exercise [113]. Additionally, the complete assessment should be conducted towards a holistic approach in order to determine individual objectives tailored to older adult’s capacities, motivation, personal needs and preferences. Delivered personalized interventions are valuable as it may improve outcomes for individuals [114]. Assessments must be followed by a post-intervention reassessment in order to highlight endpoints and adjust individual objectives. 

### 4.1. General Assessments

A CGA should be focused on uncovering deficits that may accelerate the development or progression of frailty [84]. Some approaches may be more appropriate to assess frailty accurately during a CGA. Notably, the Multidimensional Prognostic Index of frailty could predict mortality with higher performance in community-dwelling older adults than the FP index [115] and may be useful to improve the cost-effectiveness of interventions in multimorbid older adults with frailty [116]. Similarly, the Multidimensional Frailty Score was superior to predict post-operative complications at 6 months follow-up than traditional variables alone (age, grip strength, gait speed and other classifications for post-operative complications) [117]. 

In sight of exercise interventions, it remains essential to dismiss potential absolute contraindications, including: non-stabilized coronary illness, decompensated heart failure, non-stabilized arrhythmia, severe and symptomatic aortic stenosis, uncontrolled arterial hypertension (>180/110 mm Hg), aortic dissection, acute myocarditis, pericarditis or endocarditis, acute thromboembolic disease, severe pulmonary hypertension (>55 mm Hg), Marfan syndrome, non stabilized type 2 diabetes mellitus, non-stabilized orthostatic hypotension [50]. Relative contraindications should be taken on a case-by-case basis to adapt the exercise intervention in consequence: high systolic and diastolic blood pressure (>160/>100 mm Hg), coronary risk factors, chronic inflammatory rheumatisms, high risk of fall and fractures, etc. [21].

### 4.2. Physical Performances Assessments

Measurements of several physical performances often overlap within some approaches of frailty. Amongst various tests enabled for clinicians, the Short Physical Performance Battery (SPPB; a compounding score on/12 which embed the 4 or 6 m usual pace, 5-STS, and static bipodal balance) and the handgrip strength are the most used methods. Older adults exhibiting a score ≤8 may be relevant to underscore frailty [118]. Alternatively, the 30 s STS can be used for older adults who fail to perform 5-STS but also to measure fatigue [119]. Timed-up and Go (TUG) performance is also a reliable procedure to identify and discriminate frailty and may be a surrogate when full application of the FP, for instance, is unpracticable [120,121,122]. Grip strength is a strong marker of overall health and independence but its measurement should respect test-based realization conditions and be interpreted according to the device used, BMI and health conditions [2,123,124,125].

In older adults with a history of falls (at least one) within the last 12 months, frailty itself is considered as a criterion of high risk of fall, whereas pre-frail or non-frail older adults who exhibited impaired gait speed (≤0.8 m.s) or dynamic balance (TUG > 15 s) have an intermediate risk of fall [75]. Additionally, lower limb muscle power measurement seems to be relevant as it was associated with increased hazards of developing pre-frailty or frailty over four years [126]. Derived equations of the 5-STS and 30 s-STS performances can easily provide clinicians with a validated procedure to determine lower limb muscle power [119,127].

Collecting physical performances is also relevant to allocate participants to a tailored exercise program and, therefore, create homogenous group-based interventions [62,65,128,129]. Specifically, the SPPB was shown to be predictive of exercise responses and may be useful to improve participants’ outcomes [130,131,132].

### 4.3. Nutritional Assessments

The main objective when assessing nutritional status is to analyze eating habits, determine if the caloric and protein intakes covers the daily needs, and seek malnutrition in accordance with national guidelines based on the Global Leadership Initiative on Malnutrition (GLIM) consensus [133,134]. Malnutrition and frailty share common factors in their pathogenesis and are often coexisting conditions [135,136,137]. Although no longer being used for malnutrition diagnosis, the Mini Nutritional Assessment (MNA) remains widely used and strongly recommended among community-dwelling elders for assessing malnutrition (cut points ranging from <17 for malnourished older adults, ≥17 to ≤23.5 for those at-risk and >23.5 for those normally nourished) [113,138,139,140].

Unintended weight loss, often characterized by a decreased Skeletal Muscle Mass (SMM) which may lead to sarcopenia (defined by a skeletal muscle index of <5.5 kg·m^2^ in women and <7.0 kg·m^2^ in men together with a decreased in muscle strength), is prevalent among frail individuals and should therefore be measured [136]. While weight loss can be easily determined, a measurement of SMM requires advanced equipment (e.g., Dual X-ray Absorptiometry or DXA, computed tomography, D3-creatine dilution test, etc.) and lengthy examinations that are not compatible with routine care. Derived equations of SMM calculated by a Bioimpedance Analysis (BIA) represents a validated, feasible, time-efficient and cost-effective alternative, but leads to an overestimation of SMM in cases of obesity or oedema [141,142,143]. Additionally, calf circumference measurement is a suitable method to predict frailty and is positively associated with functional performances and SMM values measured by DXA or BIA [144,145,146]. Cut-points for low SMM are defined by <35 cm in men and <33 cm in women [145].

In a complementary way, the plasma concentration in vitamin D might be relevant to measure as a low level of vitamin D was associated with a higher prevalence of frailty [147]. Assessments should be followed by providing personalized nutritional counseling tailored to individual characteristics (e.g., age-related changes in appetite, comorbidities, socioeconomic status, lifestyle factors) and achievable objectives to augment the quality, the variety and the quantity of intakes [148,149]. Defining these objectives needs to take into account a higher energy expenditure for older adults who participate in the consecutive exercise program [79].

### 4.4. Physical Activity Level Assessment

The most widely used methodologies to measure PA are interviewed or self-administered validated questionnaires [150]. PA questionnaires often display poor agreement between each other and large inconsistencies in the definition of PA [151]. Only 5 of 18 PA questionnaires applicable for older adults (i.e., the European Prospective Investigation into Cancer-Norfolk questionnaire and its short form; the International Physical Activity Questionnaire-Long; the revised Morgenstern Physical Activity Questionnaire and the Physical Activity Scale for the Elderly or PASE) matched the four domains of the International Classification of Functioning, Disability and Health developed by the WHO (body function, body structures, activity and participation, and environmental factors) [151]. Moreover, sedentary behaviors and light physical activities are rarely integrated in those questionnaires which, in turn, may lead to an over or underestimation of total energy expenditure [151]. Apart from a few questionnaires used in different frailty indexes (the Minnesota Leisure Time Activity and the PASE), most of them are often time-consuming and measure PA inaccurately in a frail population. Alternatively, other approaches of PA measurement in older adults with frailty could be based on digital technologies: pedometers, accelerometers or a heart rate monitor [152]. A systematic review reported that an objective measurement of PA with wearable accelerometers discriminates frailty status and one study reported that it could help assessors distinguish the intensity of each PA behavior [153,154]. For a larger implementation, these technologies should be affordable, acceptable, feasible and easy-to-learn. These technologies need also to be studied in various settings and populations [153].

### 4.5. Additional Assessments

#### 4.5.1. Fear of Falling

Fear of falling (FoF) is characterized by a low self-efficacy or confidence at avoiding falls during essential, non hazardous activities of daily living [155]. In community-dwelling older adults, FoF was associated with an increased risk of developing frailty [156]. Measurement of FoF can be based on the Falls Efficiency Scale International (FES-I) or its short form (Short FES-I), from which scores correlate with frailty [157]. FoF should be considered with a score >23 points with FES-I and >10 with the Short FES-I [157]. Furthermore, self-reported FoF should refer to a fall severity assessment [75]. 

#### 4.5.2. Quality of Life

Frailty is associated with a worsening of quality of life (QoL) with strong evidence reported [158,159]. The short form 36 item (SF36), the abbreviated World Health Organization Quality Of Life or WHOQOL-BREF, the Quality of Life Scale or CASP-19 and the European Health Iinterview Survey-Quality Of Life or EUROHIS-QOL were reported to be the most frequently used methods to measure QoL in older adults with frailty [159]. However, these questionnaires displayed inconsistencies in the items used for QoL measurement and some are not specifically designed for older adults with frailty. Recently, Geerinck et al. (2021) proposed to use the Sarcopenia Quality of Life or SarQoL^®^ questionnaire to measure more accurately the QoL of older adults with frailty [160]. This questionnaire, which also includes a short form (SF-SarQoL^®^), was previously validated on older adults with sarcopenia and, thus, may be more appropriate and relevant to address the QoL.

### 4.6. Action Plan

Building an action plan may be essential to involve and empower older adults with frailty to change their lifestyle habits (in PA and nutrition). Proactive empowered participants are known to increase their intention to exercise and may increase self-efficacy [161,162,163] Still, there are multiple barriers to exercise participation and self-engagement in PA, notably poor motivation, perceived limitations due to chronic conditions, accessibility, affordability and concerns over negative health outcomes [23,77,164] To overcome these barriers, HCP should set specific achievable goals tailored to individual resources, capacities, competence and environment [37,76,165]. Additionally, HCP should provide knowledge acquisition in exercise and nutrition [77].

**Key points**

- Assessments of physical capacity, nutritional intake and physical activity level as part of a CGA must be undertaken to deliver personalized interventions.
- A complete assessment ensures the safety, the acceptability and the efficacy of the exercise program.
- Assessments should be followed by building an action plan to involve and empower older adults with frailty during the multimodal program.
- Action plans should be focused on personalized goal setting and provide knowledge acquisition in exercise and nutrition.

## 5. Exercise Training Components

Multicomponent exercise programs (including resistance, power, endurance, balance, functional and flexibility training) conducted to prevent and reverse frailty have to focus on individual objectives. To optimize benefits and ensure the safety of older adults with frailty, exercise programs have to be tailored, structured and progressive [21,52]. It should be noted that an exercise program may be effective for one, while being less effective or even ineffective for another [166]. Indeed, there are multiple factors that could explain an exercise response, generally classified into non-modifiable factors (genetic, age, gender, genotype) as well as modifiable factors (baseline fitness, comorbidities, polypharmacy, exercise dose, behaviors, environment, phenotype) [21,166,167]. Thus, exercise specialists should take these baseline characteristics into consideration to adjust the exercise program in order to obtain the best individual outcomes. Overall, it is considered that there are no non-responders to exercise, regarding the high and various magnitude of positive effects induced for older adults [168]. Finally, each selected intervention has to fulfill prerequisites that have been strongly established in accordance with Frequency, Intensity, Time, Type, Volume and Progressiveness (FITT-VP), generally described in this order. 

### 5.1. Frequency

The frequency is generally defined by the number of exercise sessions per week. In older adults with frailty, current recommendations indicate to perform two or three sessions per week [50,51,52,53,54,56,57]. Somes studies experimented with higher frequency in older adults living with frailty and/or sarcopenia. Indeed, recent meta-analyses showed positive improvements on several outcomes with one to five sessions per week [21,50,56,169]. Although one session per week can be sufficient to improve strength and physical performances, greater gains on muscle strength and muscle size are induced with two to three sessions per week [50,51,61,170]. However, attending more than three exercise sessions per week may be difficult for older adults with frailty, as suggested by authors, and it may interfere with adherence [27,43,47].

Defining frequency should embed rest periods, especially in older adults with frailty. Indeed, the process of muscle regeneration following exercise-induced mechanical stress by satellite cells is impaired by prolonged periods of inactivation; in other terms, sedentary, a common adverse behavior in older adults with frailty, may reduce the ability to recover from exercise sessions [171,172]. In the beginning of an exercise program, sedentary older adults with frailty may also experience Delayed Onset Muscle Soreness (DOMS). Thus, rest periods are often expected by older adults with frailty to recover from DOMS and fatigue. As a result, it is recommended to set non-consecutive exercise sessions and leave at least one day of resting [57,173]. It is important to underline that multimorbid frail individuals, notably with sarcopenia, might require longer rest periods [68].

In-between sessions should be stimulated by self-engagement in PA, minimally, to reduce the time spent in sedentary activities [11]. To this end, exercise specialists should foster involvement by stimulating simple and feasible physical activities of daily living (e.g., walking, shopping, garden and household activities, etc.). This stimulation should take into account individual physical and cognitive capacity, chronic conditions, motivation, social support and environment. 

### 5.2. Intensity

Intensity is one of the key components (with volume) to induce training-related adaptations (i.e., strength) and, thus, its adjustment may optimize individual outcomes [57,60]. Although low-intensity exercise programs demonstrated benefits, comparative studies showed that moderate-to-vigorous intensity exercise programs produced greater outcomes on strength in healthy and older adults with frailty [50,61,174,175,176]. Progressive moderate-to-vigorous intensity exercises tailored to individual capacity, limitations and comorbidities have also demonstrated their feasibility, acceptability and safety [21,177]. However, it remains important to gradually modulate intensity in order to ensure proper exercise tolerance, avoiding adverse events (injuries, DOMS, etc.) and potential drop-outs [21,50]. Finally, moderate-to-vigorous intensity should be set on exercises being mastered by participants, notably to avoid the risk of fall and fractures [50,178].

For resistance training purposes, intensity is generally considered as the training load, expressed in percent or in absolute value, relative to maximal dynamic strength known as the “One Repetition Maximum” or 1RM [50]. Generally, the 1RM evaluation in older adults is measured by submaximal measures or estimated by specific equations [179]. Because 1RM evaluation protocols are not easily implementable in older adults with frailty, an increasing number of authors are using Rating of Perceived Exertion (RPE) scales (i.e., Borg scale) that have been validated in older adults to monitor intensity [54,180,181,182]. Perception of effort is a subjective self-reported perception and is defined by how a physical task is hard, heavy and strenuous [183]. The most commonly validated and used scales are Borg Category-Ratio 10 (CR10); Borg CR100 and Borg 6-20. If used correctly, these scales offer a feasible alternative to monitor intensity and studies showed that Borg scales correlate moderately with the 1RM [184,185,186]. Other approaches include Repetitions in Reserve (RiR; i.e., exerting until one repetition is left in reserve) or velocity monitoring (i.e., exerting until reaching 10% loss of velocity of the exercise performed) [187,188]. Interestingly, while it has been established that RM, RPE, %1RM and RiR were equally effective to improve physical performance in older adults, RPE appears to be the most tolerable and enjoyable method. Thus, it may be recommended in older adults with frailty [67].

In practice, exercise programs are generally based on intensities ranging from 20% to 50% of 1RM (Borg CR10 to 2-3/10) in the initial phases of training, progressing to 70–85% 1RM (Borg CR10 to 8/10) [27,43,50]. This progressive approach has proved to be effective for a wide range of physical and functional performances (muscular strength and power, gait speed, etc.) [27,50,175,176]. Strength and functional improvements induced by moderate-to-vigorous intensity resistance training are firstly associated with muscle reinnervation, an increase in motor units discharge, fiber-type-specific myonuclear adaptations, type IIa fiber shifting and mitochondrial remodeling [189,190,191]. To achieve significant body composition improvements (i.e., increase in muscle mass), it may be necessary to use higher intensities (70–85% of 1RM) than those required to achieve significant improvements in strength (45–80% of 1RM) [51,191]. The main physiological-related cause may lie in architectural adaptations to exercise training, characterized by mechanical overload and activation of specific molecular pathways which, in turn, increase muscle cell growth and, thus, increase the muscle cross-sectional area [56]. This mechanistic explanation has reinforced the importance of progressing the intensity, as it was already associated with an increase in muscle protein synthesis [54]. In contrast, power training should be performed at lower intensities from 30 to 60% of 1RM [50].

Both in session and in between endurance activities should focus on light-to-moderate or moderate-to-vigorous intensities [192]. Exercise specialists can refer to MET (computing resting energy expenditure equivalent of 3.5 mL·min^−1^·kg^−1^ of oxygen consumption) to guide older adults in such PA in order to achieve goals close to the WHO recommendations (i.e., at least 150 min of moderate intensity PA per week) [193]. To this end, moderate intensity PA can be computed by MET, encouraging PA between ≥3.0 and ≤6.0 METs.

### 5.3. Time and Rest Periods

For older adults with frailty, meta-analyses and guidelines agreed upon a minimum of 12 weeks of training to improve strength, functional and balance outcomes [27,53,54,55]. Indeed, authors observed improvements in strength during the first 3 months of moderate-to-vigorous activity, induced by rapid neuromuscular adaptations [189,194,195]. However, a longer duration combined with an increase in intensity and volume (i.e., 16 weeks) could favor greater outcomes and, thus, may increase responder prevalence [168,196]. Borde et al. found consistent evidence as the longest exercise program analyzed was also the one with the greatest increase in strength [51]. Nonetheless, 6 to 9 weeks may be sufficient to induce strength improvements in healthy older adults, but those with frailty may require longer duration to have substantial gains [27,51,196].

The duration of an exercise session is about ~1 h in order to achieve a complete multicomponent session (strength, power, endurance, functional, balance and flexibility) [17,53,56,65]. From a practical point of view, resistance training should be incorporated first, balance and functional training second and endurance activities last [21]. Prior to each exercise session, a complete warm-up of 10 to 15 min including cardiovascular, respiratory and musculo-articular movements must be integrated [197]. Resistance-based exercises should last 20 to 30 min to induce sufficient adaptations as it is recommended to set 2–3 exercises per major muscle group per day [43,50]. Balance exercises should last approximately 10 min. Endurance activities should last 5 to 10 min and should progress to 15–20 min [21,43]. At the end of each session, 5 to 10 min of low-intensity stretching exercises (i.e., passive stretching without discomfort) should be included as part of the cool-down phase [43].

Shorter forms, such as circuit resistance training, might also be of interest in regards to its advantage to improve muscle strength with time efficiency (around 40 min) at lower intensities (i.e., modest: ~40–60% of 1RM), with reduced resting time between exercise sets (10–30 s or no rest vs. 1 to 2 min) [198]. However, this approach included non-frail older adults with a younger age (mean 64.5) and requires specific adaptations to comorbidities (i.e., cardiovascular diseases, etc.) to manage exercise tolerance [50]. Other time-efficient approaches, such as high-intensity interval training, are safe and well-tolerated but provide limited evidence in older adults. Thus, it may not be recommended as a single approach and should be implemented with caution in older adults living with frailty [199].

The rest time was also presented to have influences on muscle strength and muscle mass gains. Overall, it is recommended to set 1 to 2 min of resting intervals between exercises [59]. Interestingly, a systematic review found a tendency toward significance for 60 s of rest between sets that produced the largest effect on muscle strength [51]. In adults, shorter rest periods (i.e., 30 to 60 s) between exercise sets may result in greater exercise-induced circulating anabolic hormones and, thus, may produce hypertrophic gains [200]. However, this phenomenon remains unknown in older adults with frailty [200]. 

The time under tension per repetition should be taken into consideration by exercise specialists as it is involved in the various neuromuscular adaptations to training, notably in the recruitment of motor units and their firing rates. Borde et al. (2015) showed that the most effective time under tension to improve strength appears to be 2.5 s for concentric, 2.0 s for isometric and 3.0 s for eccentric contractions [51].

Overall rest time should be determined in accordance with exercise tolerance and RPE [68]. This is particularly important at the very beginning of exercise programs to avoid excessive fatigue, pain and potential injuries. Furthermore, sufficient rest time should also be necessary to bring corrections upon proper techniques to adopt [57]. In early training phases for older adults with frailty, exercise specialists should give closer attention to rest time rather than the number of sets per exercise, as volume was not the primary variable responsible for muscle strength improvements [50].

### 5.4. Type

Multicomponent exercise programs including resistance (i.e., strength and power), endurance, balance, functional and flexibility training are the most effective interventions for older adults living with frailty to induce substantial improvements, notably in muscle strength and function [21,27,28,39,55]. This may be explained by the different intrinsic adaptations related to each type of exercise [50]. During such programs, emphasis should be placed on functional-based resistance and power training [21].

Strong evidence supports the benefits of resistance training in strength, functional and balance outcomes, and with low-to-moderate evidence in body composition [63]. Resistance-based training is recognized as a reference method to tackle neuromuscular impairments with aging and enhance myofiber hypertrophy, fiber shifting to type IIa, motor unit efficacy, muscle protein synthesis, neuromuscular performances and functional capacity [92,167]. Moreover, resistance-based training leads to additional significant improvements in cognition, fall risk, functional independence, quality of life, self-efficacy and reduces several chronic disease risks [18,50,64,163,201,202,203]. Progressive power training integrating generally high velocity concentric contractions is also crucial given the close relationship between muscle power and functional activities of daily living [21,67]. In older adults with frailty, increasing the evidence of power training demonstrated significant improvements in muscle power output and functional performances to prevent the incidence of falls [42,204].

Interestingly, studies have demonstrated that resistance-based training induced some similar adaptations related to endurance training (i.e., increase in mitochondrial density, oxidative enzymes and cardiovascular function), whereas comparative studies showed that endurance training alone led to much more modest improvements, or even no improvements at all in strength and hypertrophy outcomes [205,206,207]. However, endurance training remains essential to improve cardiorespiratory fitness and function (i.e., maximum oxygen consumption peak or VO^2^ peak), as well as to reduce cardiometabolic risk factors [208]. More specifically, combined exercise training (endurance and resistance) is recommended in obese older adults with sarcopenia with the objective of reducing the percentage of fat mass while preserving muscle mass [209,210].

In addition to resistance training, dynamic balance and functional training is important to improve autonomy for daily living and functional performances, and reduce fall risk [50,64]. Adapted physical and sport activities, such as dance, may also present an interest to enhance balance, social participation, well-being and adherence in older adults [211,212]. However, the dose-response relationships of balance exercises remain undetermined [213]. Balance exercises should not be predominant over resistance training. Flexibility training may also be recommended to increase the joint’s range of motion and gait performance in older adults with frailty [43,66]. However, evidence for functional outcome improvements remains scarce and controversial [27,214]. Finally, adding a cognitive intervention to the exercise program (separately or as part of the training components) may be useful to improve cognitive performances, especially of executive functions [215]. 

New technologies offer opportunities to implement interactive types of exercises such as exergames; remote home-based virtual exercise sessions and virtual reality gaming. In older adults with frailty, systematic reviews reported that exergames are safe and showed good adherence rates [216]. Exergames alone have been shown to improve mostly balance and functional outcomes but not strength [216]. Interestingly, exergames may foster PA engagement as a higher level of intrinsic motivation was observed in several exergaming interventions [217]. However, poor evidence is reported and high quality studies are required, as well as standardized protocols [216,217,218,219]. Remote home-based virtual exercise sessions were continuously developed during the COVID-19 pandemic and demonstrated significant improvements in function, psychological well-being and balance [220,221]. Between supervised exercise sessions, this intervention may be interesting to support PA maintenance and avoid decline in functional status [221]. Lastly, virtual reality gaming demonstrated benefits in older adults with a high risk of fall, improving balance and physical function following an intervention of 5 to 8 weeks, ≥3 sessions per week of 20 to 45 min [222].

Other evidence-based types of physical activities that may be beneficial for older adults with frailty include tai-chi, yoga, Nordic walk or whole body vibration. Poor-to-moderate evidence is reported for yoga and tai-chi interventions to improve physical function, strength and muscle mass [44,223]. However, yoga and tai-chi may be useful to improve psychological outcomes, notably, fear of falling, anxiety, self-efficacy and well-being [44,45,224]. Nordic walk may be interesting in older adults with osteopenia or osteoporosis as it has been shown to improve body composition, knee extensor strength and functional capacity [46]. Whole body vibration, associated with conventional resistance training, does not demonstrate any additional improvements but may be interesting in older adults with disease-related functional disabilities (e.g., hemiplegia, etc.) [169]. Overall, accurate protocols and dose-response relationships remain to be determined before selecting one of these practices as a single method.

### 5.5. Volume

In resistance training, volume is considered to be the absolute quantity of exercise performed during a session (i.e., sets × repetitions) [50]. Overall, it is recommended to perform a progressive volume ranging from 1 to 4 series to 6 to 15 repetitions [50,52,56,57]. Volume could be used as an indicator of training intensity [51]. Hence, volume is an important determinant for training-related adaptations [50,51,58]. Indeed, although older adults experienced less increase due to a blunted anabolic response, higher volume was significantly associated with greater gains in muscle mass in a previous study [58]. However, dose-response relationships in terms of the increase in muscle mass remain unknown as an insufficient number of studies were identified in the literature [51]. Still, in healthy older adults, effects on muscle morphology may be expected with a volume of 2–3 sets of 7 to 9 repetitions, performed at 51 to 69% of 1RM, during 50–53 weeks, 2 to 3 sessions per week, and with a rest time of 2.5 s between repetitions and 120 s between sets [51]. In older adults with frailty, volume augmentation must be in accordance with a subsequent decrease in intensity and have to be in line with the principles of individualization and exercise tolerance [21,50]. Setting repetitions until failure does not seem necessary as it does not influence neuromuscular adaptations [50]. Alternatively, older adults may benefit from a strategy consisting in lowering the exercise volume, together with an inverse relationship in intensity and frequency [225]. This minimal-dose strategy may be an interesting surrogate to overcome the traditional increase in volume that could be difficult to reach at some points. Furthermore, it may help to implement a sustainable strategy toward PA across the lifespan. Although being a promising strategy, several questions remain unanswered and this strategy needs to be investigated on frailty.

Conversely to resistance training volume, a dose-response relationship of the global PA volume has been highlighted. Indeed, older adults meeting WHO guidelines (i.e., 150 min of moderate-to-vigorous physical activities per week) had less frailty and better physical function [72]. However, for frailty management, other authors acknowledged that 150 min of moderate-to-vigorous physical activity per week may be unreachable for older adults with frailty [11]. Although higher doses of PA are required to obtain significant improvements in QoL (180 min per week), emerging evidence suggests that less than 150 min may be sufficient to induce positive results on physical function and mortality [226,227]. Indeed, among inactive older adults, less prolonged sedentary time and, thus, more light PA has been associated with lower frailty scores [10]. Moreover, data showed that non-prolonged sedentary time (<30 min per day) does not correlate with mortality risk in older adults with frailty, suggesting a protective effect of breaking sedentary time by light PA [11]. Similarly, in a 3 year observational study, Zhao et al. demonstrated that daily walking time (30 min to 1 h per day) displayed a greater association with decreased frailty risk [228]. Nonetheless, a higher dose-response effect seemed to be necessary to reduce mortality, in this case 375 min of light physical-activity per week [12]. In contrast, a smaller dose-response of moderate-to-vigorous PA (about 24 to 27 min per day) was necessary to offset the detrimental effects (notably mortality) of sedentary time on frailty [10,12]. During the follow-up of an exercise program, the most active quintile of older adults maintained their physical performances to a greater level and, thus, prevented the onset of mobility disability [14]. These benefits were induced with a relatively small and achievable dose-response effect with an additional 48 min per week of PA from the baseline volume of PA [14]. Regardless of intensity, the first step of self-engagement in PA should be focused on involving older adults to reduce sedentary behaviors by increasing light PA volume (e.g., increase in daily steps if pedometers are implementable). 

New technologies may help older adults to monitor their PA level, in particular wearable pedometers. In older adults with frailty, Watanabe et al. observed a significant reduction in mortality from >5000 steps per day but it may be difficult to reach, as asserted by authors [229]. Instead of aiming for 5000 steps as a first objective, it is suggested that adding 10 min per day (~1000 steps) of moderate-to-vigorous activity may be achievable and relevant. Indeed, this behavioral change was associated with several positive outcomes (i.e., in mortality, chronic disease incidence and dementia risk) [230].

### 5.6. Progressiveness

Progressiveness refers to a gradual and systematic increase in workload (i.e., stress placed on the body) to induce continual training adaptations over time [68]. Generally, a progressive increase in workload includes an increase in intensity or in volume to optimize adaptations [50]. The concept of progressiveness takes the principle of individualization into account as older adults with frailty may benefit from an exercise program starting at an appropriate level tailored to their physical and cognitive capacity, medical conditions, motivation and goals before progressing to advance phases [50,175,176,231]. As it is generally admitted that neuromuscular and morphologic adaptations have different temporalities, training progressiveness could be divided into different phases embedded into a periodization process [50,70,189,194,195,232]. A meta-analysis showed that periodized exercise programs were superior to non-periodized exercise programs to induce strength gains, regardless of gender and age differences [233]. Conversely, Conlon et al. stated that periodized programs do not seem essential in older adults as no differences between periodized and non-periodized programs were observed in untrained individuals (mean age 72.9) [234,235]. Nonetheless, time-block programs might be interesting and feasible in older adults living with frailty, according to studies that used this strategy [132,181,236]. Overall, these programs embed a familiarization phase of 2 to 4 weeks at 20 to 50% of 1RM. Sedentary older adults with frailty might benefit from this strategy to increase their self-confidence, learn proper techniques, avoid DOMS and, thus, might help to increase their adherence [50,231]. As data are relatively scarce in this field, it remains difficult to conclude in favor of periodized exercise programs for older adults with frailty.

**Key points (see Table 1).**

- Conception, elaboration and implementation of a tailored multicomponent exercise program for older adults with frailty have to integrate appropriate frequency, intensity, type, time, volume and progressiveness to warrant individual benefits.
- Program has to be structured around 2 to 3 non-consecutive sessions per week of ~1 h each, at a progressive low-to-moderate-to-vigorous intensity of 20 to 85% 1RM or RPE 2 to 8/10, for a minimum of 12 weeks, with an emphasis on functional resistance training (strength and power) and endurance.
- Progressive volume has to range from 1 to 4 sets of 6 to 15 repetitions, with a rest period of 1 to 2 min depending on exercise tolerance and chronic conditions.
- Between exercise sessions, older adults with frailty have to be encouraged to increase the PA volume of light–to-moderate activities to maximize outcomes, minimally reducing time spent in sedentary activities.
- Daily life PA has to be stimulated with step-to-step personalized and achievable goals tailored to physical and cognitive capacity, motivation, comorbidities and environment.

## 6. Exercise Program Contents

Conception, elaboration and implementation of a complete exercise session requires a precise construction that should be time-efficient, customizable and progressive to also deliver an appropriate training stimulus (i.e., what refers to overload). 

### 6.1. Exercise Selection

Emphasis should be placed on lower body muscles regarding their higher age-related decline and their crucial role for performing functional activities of daily living [128]. Increasing strength of the thigh appears to be a central outcome as femoral muscles are a major contributor to physical function [237]. Moreover, maximum isometric strength of knee extensors was associated with fall risk in very old adults (mean age 88 ± 7 years) [238]. Plantar flexor strength and ankle mobility are important as they are associated with dynamic balance, static balance and functional capacity [239,240]. In cases of falls, hip and knee flexors are decisive to help older adults get up from the floor independently and should be strengthened to this end with additional knowledge on proper techniques to adopt [69,202]. Thus, strength and functional training of the quadriceps, hamstrings, iliopsoas, gluteal, plantar and dorsi flexors should be prioritized. Numerous lower limb exercises can be included, as long as they are adapted to participants’ capacities, limitations and tolerance. In the early phases of training, it is preferable to include multi-joint movements at bodyweight prior to mono-joint movements with additional resistance, given the close relationship of multi-joint exercises with functional capacity [50,57,64]. For instance, efficient resistance- and functional-based exercises should incorporate sets of squats; sit-to-stands (or leg press if machine-based); lunges; knee extensions; leg curls or plantar and dorsal flexions.

Upper-body adapted resistance exercises are significant to improve grip and trunk strength, both playing a role in maintaining independence for activities of daily living [68]. One study reported that core strength training could alleviate age-related deficits in trunk strength, improved functional performances, balance and reduced falls with small-to-medium effects [241]. Task-specific handgrip exercises should also be included to optimize gain in grip strength [242].

Endurance activities for older adults with frailty can include walking, treadmill walking, ergocycling, step-ups, stair climbing or adapted sport and physical activities. The choice of these activities depends on the feasibility, individual capacity, medical conditions and individual preferences [21]. 

Balance exercises can be grouped in gait retraining (e.g., walking with change pace, tandem walking, heel-to-toe walking, etc.), notably with dual-task exercises, which is an interesting modality to improve cognitive capacity [243,244]. 

Flexibility exercises should focus on constant angle stretching at a low intensity. Studies in adults and athletes agreed upon a progressive volume of no more than 60 s per muscle group to improve range of motion and avoid a decrease in maximal voluntary strength and power induced by a longer time [245]. In a comparative study, Sobolewski et al. reported that repeated 4 sets of 30 s increased the passive range of motion similarly between young and older adults [246]. In view of disparate protocols reported in the literature, it is difficult to recommend flexibility exercises that focus on specific muscle groups. Additionally, while no more than 60 s of tension appears to be relatively sufficient to induce improvements in adults, avoiding high-intensity stretching movements (i.e., when participants feel pain) may be important in older adults with frailty. Indeed, it might be associated with subsequent increased inflammation in participants with chronic conditions [247].

### 6.2. Exercise Regimens

Resistance-based exercises can be performed with different tensions (concentric, eccentric and isometric), each having specific advantages. Concentric contractions should be prioritized as concentric and isometric forces decrease twice more during the aging process than eccentric force [248,249]. However, changing contraction regimens to eccentric and/or isometric may be interesting to induce various adaptations on muscle [248,250]. The eccentric method is associated with multiple advantages, notably, less cardiovascular stress, lower oxygen consumption and, thus, lower RPE relative to higher loads [251,252]. Although studies reported that eccentric-based training did not demonstrate any superior strength improvements in lower limbs compared to concentric training at constant velocity, exercise specialists should consider its clinical relevance for older adults with frailty or those with chronic health conditions [71,252,253,254]. Indeed, eccentric training demonstrated greater gains in older adults with frailty on muscle strength, balance and hypertrophy, together with a reduced energy cost, compared to a conventional concentric resistance training [255]. Another argument is that eccentric force is relatively preserved in older adults and, thus, eccentric training offers a physiological advantage to induce substantial improvements [248,249,253]. Lastly, older adults may benefit from eccentric training to improve functional activities of daily living, notably stair climbing and descent [254,255]. Although eccentric training is feasible (does not necessarily require advanced equipment) and safe, it should be noted that premature eccentric training without familiarization beforehand may increase the likelihood of DOMS [253,254,256]. Eccentric exercises should therefore be performed at 4 to 8 weeks of an exercise program, given that eccentric and isometric neural activation increases were observed at this temporality [195].

Additionally, isometric training is known to provide a safe base for dynamic balance exercises but also for activities of daily living [70]. It also represents an interest in cases of joint discomfort or pain in order to progress to a tolerable range of motion, notably in older adults with arthritis or musculotendon-related pain [21,70].

**Key points**

- Exercise selection has to emphasize lower limb exercises, in particular those recruiting femoral muscles, complemented by additional exercises of the upper body (especially grip and trunk).
- Priority should be be given to functional multi-joint exercises to improve physical function.
- For older adults with frailty, functional multi-joint exercises at bodyweight should be included at first before progressing with additional resistance.
- Overall, concentric contractions should be predominant over eccentric, but progressing to eccentric contractions should be considered as a safe and relevant method to improve training performance and physical function.

## 7. Intervention Settings

### 7.1. Free-Weight or Machine-Based?

Engagement in an exercise program remains challenging for older adults with frailty due to intra-interpersonal and environmental factors [23,77,164]. Therefore, practical applications have to be feasible and acceptable to foster individual adoption. Generally, two distinct approaches meet these requirements and could be used to implement an exercise program: resistance-based with small equipment or weight-bearing; and resistance-based with machines. Although these approaches induce relatively similar results on muscle strength, power, function and balance, specific advantages should be taken into consideration for program implementation [175,257,258,259]. In older adults with mobility impairments and/or a limited range of motion, machine-based resistance training is generally recommended as both familiarization and safety are made easier [50,57]. Gain in muscle mass might be increased with machine-based training, but evidence remains poor [260]. However, advanced equipment poses a problem of accessibility and adaptability for older adults with frailty.

Resistance-based training with small equipment (dumbbells, elastic bands, ankle weights, etc.) represents an adequate alternative in terms of safety, feasibility and replicability for older adults with frailty [62,68,261]. In adults athletes, it was shown that muscle electrical activity was similar between machine-based and free-weight bench press, and was somehow higher in free-weight at specific intensities [262,263]. With small equipment, older adults could easily replicate movements at home to simulate activities of daily living [257]. With regard to additional muscular activity required to control movements in this approach, close attention should be paid to proper techniques to avoid risk of injury [57]. As such, a familiarization phase of 1–2 weeks should be included prior to increasing training stimulus [50]. In this phase, some exercises could be performed in seated positions, especially in mobility-impaired older adults [50,68]. The use of unstable surfaces (e.g., Swiss balls, Both Side-Ups or BOSU balls, etc.) could also be of interest if supervised. In healthy older adults, it has been shown that this approach is feasible and as effective as machine-based training to improve lower limb strength, power and balance [258].

To summarize, exercise-induced improvements appear to be independent of practical approaches. It is assumed that exercise-induced improvements are, rather, the results of the exercise training method. From a prevention perspective for older adults with frailty, HCP should implement an exercise program that is feasible, acceptable, easily accessible and replicable.

### 7.2. Unsupervised Home-Based or Supervised Group-Based?

Choosing between home-based or group-based settings is highly relevant for adherence and motivation. In older adults aged 50 to 65 years without frailty, studies found a significantly higher adherence rate in unsupervised home-based exercise programs than in group- or center-based settings [264,265]. In contrast, in higher ages (>65) and with the presence of frailty or sarcopenia, adherence is generally higher in group-based settings [181,266]. In addition, group-based settings appear to be superior to improve physical performances, reducing frailty status [20,267] and might be of interest in rural areas [268]. Besides beliefs and individual preferences, group-based settings may embed several interpersonal factors predictive to exercise engagement and maintenance. Indeed, qualitative studies reported that self-reliance, social bonds, peer support and exercise learning influence self-efficacy, which is highly determinant for long-term PA maintenance in older adults [76,77,269,270]. Additionally, group-based settings may support involvement and empowerment to promote self-efficacy [163], while home-based settings seem to induce only short-term improvements that were lost after follow-up [271]. In this perspective, supervised training appears to be essential and should be delivered by highly competent exercise specialists (e.g., kinesiologist, etc.). Indeed, a lack of professional guidance was considered as a main barrier for older adults before participating in an exercise program [272]. Moreover, a systematic review demonstrated that supervised exercise programs for older adults are superior to improve balance, power and strength outcomes in comparison with unsupervised programs [74]. However, tailoring allocation to either group- or home-based training to individual preferences remains important as some older adults may perceive group-based training as a barrier and rather prefer to practice alone [272]. HCP should therefore define a compromise between targeted individual outcomes and individual preferences in order to upvote program settings. Yet, systematic reviews and meta-analyses reported a high quality of evidence in favor of supervised group-based exercise programs for older adults with frailty [20,273].

Although group-based settings offer numerous advantages, several practical limitations have to be highlighted. Some regions face socio-territorial inequalities in terms of population characteristics (socio-economic status, socio-cultural, etc.), availability of specialized HCPs and accessibility to facilities. Thus, home-based exercise programs could represent an interesting alternative. Specifically, fall prevention could benefit from home-based settings as older adults at-risk would rather practice an exercise program at home or close to it [274]. Randomized Controlled Trials (RCT) reported that home-based exercise interventions with supervision are feasible and effective to reduce subsequent falls [275] and improve functional abilities [271]. Moreover, a systematic review concluded that home-based exercise programs are simple, safe, widely implementable and can prevent frailty, in particular in the pre-frailty state [276,277]. Finally, home-based exercise programs targeting participants aged ≥80 years with a high risk of falls may reduce health-related costs [278]. However, supervised exercise programs may save money to a greater level [279]. As a result, the supervision of home-based exercise programs for frailty management may be more suitable. Several studies show that unsupervised programs did not improve physical outcomes to a greater level, compared to supervision [73,276,280]. Thus, unsupervised home-based training may only be indicated for robust or pre-frail older adults without chronic conditions that would impose exercise adaptations (i.e., heart failure, diabetes mellitus, osteoporosis, etc.) [73]. Still, robust older adults improved their physical performances to a greater extent when participating in a virtual exercise program delivered in live, supervised sessions than in unsupervised recorded sessions [281]. In multimorbid frail individuals with severe functional impairments (i.e., SPPB < 5) and a high risk of fall, individual tailored interventions that are not only focused on exercise should be recommended [38,65,75].

**Key points**

- Exercise intervention settings have to be in line with feasibility, acceptability, accessibility and replicability.
- Advocate resistance training based on free-weight and small equipment.
- For older adults with frailty and/or chronic health conditions, supervised group-based interventions are recommended as they provide several advantages that can foster involvement, empowerment and self-efficacy.
- Unsupervised or remote home-based exercise programs are safe and effective but should be more indicated to robust older adults or those with pre-frailty.

## 8. Behavioral Strategies

In order to elicit sustainable changes in PA behaviors outside supervision, strategies should be focused on specific goal-setting behaviors, self-efficacy, intrinsic motivation for PA, outcome expectancies and continued peer-social support [76,78]. Increasing older adults’ intrinsic motivation for PA (i.e., satisfying their basic psychological needs: autonomy, competence and relatedness) may be critical as it was associated with better adherence, higher quality of life and improved body composition [282,283,284,285]. To foster self-determination in older adults with frailty, Ekelund et al. proposed to establish a person-centered approach: give them opportunities to feel involved and improve their health literacy and their confidence in social relationships (i.e., with family and caregivers) [35]. Additionally, older adults with frailty changing their behaviors may benefit from HCP who offer support, counseling and follow-up [77,272]. Embedding a behavioral-like strategy showed meaningful outcomes in older adults with frailty, as one RCT reported higher compliance and greater improvements on mobility and frailty status [33]. Behavioral strategies showed significant long-term benefits on physical outcomes and PA level, and may prevent detraining effects that, notably, occur rapidly in older adults with frailty following a supervised exercise intervention [39,286,287].

**Key points**

- Behavioral strategies are strongly advocated to elicit sustainable changes in PA and prevent detraining effects.
- Behavioral strategies should include specific goal-setting behavior, self-efficacy, intrinsic motivation for PA, outcome expectancies and continued peer-social support.

## 9. Nutrition

A balanced diet is essential for global health and musculoskeletal function [288]. Covering caloric and protein needs with sufficient intake is necessary to maintain muscle mass and function [79]. Interestingly, certain proteins appear to be of specific interest: leucine, citrulline, creatine and Hydroxy-Methyl-Butyrate (HMB). While the influence of proteins remains the most studied, other nutrients such as vitamin D, omega-3 fatty acids and antioxidants also seem to have an influence on muscle parameters.

### 9.1. Proteins and Amino Acids

Protein intake needs are generally higher in older adults [289]. The European Society of Clinical Nutrition and Metabolism (ESPEN) recommends at least 1.0–1.2 g protein/kg body weight/day for healthy older adults and 1.2–1.5 g protein/kg body weight/day for older adults with chronic or acute illness [80]. An association between low protein intake and sarcopenia is currently accepted, as shown by the results of recent meta-analyses on muscle mass and physical performance [81,82]. Among fast proteins, whey protein derived from milk appears to stimulate myogenesis more than casein derived from other protein sources [290]. Essential amino acids (EAA), particularly leucine, stimulate anabolism [291]. The main dietary sources of EAAs are lean meats, dairy products, soy, peas and lentils. HMB is a leucine derivative with an anti-catabolic effect on myoproliferation and myodifferentiation [292]. Its concentration decreases with age as leucine metabolizes less and less efficiently into HMB [80]. Its supplementation in older adults may therefore be rational and, thus, may help to prevent the onset of sarcopenia. According to a meta-analysis, leucine increases muscle mass, but this effect was only observed in older adults with sarcopenia [293]. Global concerns about environmental sustainability have led to a growing interest in plant-based proteins [294]. Care should be taken, however, as not all plant protein sources contain all the essential amino acids that animal sources do. Therefore, it may be important to vary plant protein sources and consume large quantities to cover all amino acid requirements [295].

### 9.2. Vitamin D

Low vitamin D levels are predictive of an increased risk of sarcopenia [296,297]. Recent research has suggested that vitamin D may also trigger skeletal muscle hypertrophy and promote protein synthesis via the Mamalian Target of Rapamycin (mTOR) complex 1 signaling pathway [298]. Vitamin D supplementation in highly deficient older adults with a threshold of less than 25 mmol/L has a beneficial effect on sarcopenia indicators, while there was no effect in non-deficient subjects [299]. A dose of 600–800 International Units (IU) per day is recommended, targeting a circulating level of 75 nmol/L in older adults [300]. The association of vitamin D and leucine-enriched whey protein has shown results in muscle mass and lower-limb function in older adults with sarcopenia [301,302].

### 9.3. Omega-3 Fatty Acids

Consumption of oily fish appears to have a positive effect on frailty status in adults, but this effect progressively disappears in older adults (at ages > to 70) [303]. A meta-analysis indicates that omega-3 supplementation has positive effects on muscle mass but also on quadriceps strength when exercise was combined [304]. Similarly, supplementation with omega-3 fatty acids (with a daily intake of >2 g per day) may contribute to muscle mass gain, but evidence is poor and interactions with exercise remain unknown [305]. As a result, more studies are needed before this supplementation could be recommended [306].

### 9.4. Antioxidants

Antioxidants help to reduce oxidative stress that may underlie, among other factors, sarcopenia [307]. Molecules with antioxidant properties, such as polyphenols and curcumin, are of particular interest. Interestingly, a recent study has suggested a possible muscle-specific response to curcumin treatment in aging mice [308]. However, the effect of antioxidant supplementation on muscle performance is still largely controversial and should be provided with caution. Indeed, excessive antioxidant supplementation in non-antioxidant-deficient individuals may compromise the exercise-induced antioxidative effect and, thus, may lead to less outcomes [309]. Overall, systematic supplementation is not recommended but instead promoting regular consumption of foods naturally rich in antioxidants may be beneficial in older adults [310].

### 9.5. Gut Microbiota

A recent review concluded the importance of the intestinal microbiota, known for its actions on energy balance and inflammation [311]. Aging is associated with intestinal dysbiosis and a more permeable intestinal barrier, leading to the passage of inflammatory compounds. This systemic inflammation may be a predisposing factor in sarcopenia [312]. The intestinal microbiota is thought to play a role in physical exercise adaptations [311]. Interventions focusing on the intestinal microbiota-muscle axis may be a useful means to counteract sarcopenia [311]. However, it should be stressed that evidence for the efficacy of prebiotics, probiotics and symbiotic comes mainly from animal studies [313,314,315].

### 9.6. Mediterranean Diet

The Mediterranean dietary pattern is characterized by a high consumption of fruit, vegetables, a variety of plant-based foods, the use of olive oil and a lower consumption of meat and dairy products. The Mediterranean diet helps to provide an increased intake of micronutrients and other bioactive compounds. Adherence to diets combining different nutrients in a more or less complex manner seem to have a positive and long-term role on muscle parameters [316,317]. More recently, adherence to the Mediterranean diet has been found to be associated with a lower prevalence of sarcopenia [318]. Still, evidence remains poor in this field.

**Key points**

- Balanced diet covering caloric and protein demand that also provides essential nutrients is crucial to prevent sarcopenia and frailty.
- Vitamin D, omega-3 fatty acids and antioxidants should have special considerations in the diet habits.
- Evidence for nutritional interventions alone to improve sarcopenia outcomes does not support their implementation.

## 10. Exercise with Nutritional Interventions

Although most epidemiological data attest to a relationship between nutrition and frailty, nutritional interventions alone remain insufficient to prevent frailty and sarcopenia, a geriatric syndrome closely related to physical frailty [3,13,319]. Nonetheless, nutrition may play an adjuvant role in resistance-based exercise interventions in the prevention of sarcopenia [29,30,82,320].

### 10.1. Proteins and Amino Acids

When combining exercise and nutrition, greater musculoskeletal benefits are likely to be achieved with optimal nutrition (including mostly proteins) to stimulate muscle adaptations and outcomes [321]. Indeed, recent meta-analyses observed that exercise with nutrition performed best in reducing 5-STS time [29,320]. However, data remain controversial on physical performances as meta-analyses showed a larger effect on grip strength when combining nutrition [29,322], whereas another did not [320]. Still, exercise interventions alone are largely sufficient and superior to improve physical performances and quality of life in older adults with sarcopenia, with moderate-to-high evidence reported [29,322]. Nutritional interventions may also enhance gains in lean body mass induced by resistance exercise [302,320,323,324]. Older adults with sarcopenia may likely witness greater effects on lean body mass and lower body strength with a protein ingestion of 1.6 g/kg/day [325]. However, the anabolism response may be blunted in older adults and, thus, may impede nutritional adjuvant effects on muscle mass [326].

The combination of protein/amino acid administration with exercise has been shown to have a synergistic effect on increased muscle protein anabolism, compared with each intervention alone [322,327]. The combination of creatine supplementation and resistance training increases insulin-like Growth Factor-1 (IGF-1) at muscle level [328], resulting in increased muscle mass and strength [329]. Moreover, creatine supplementation combined with progressive resistance training showed large effects on muscle mass and strength [82]. However, no clear effect was reported on physical performances [330] as not all studies showed a benefit in terms of muscle strength with the use of creatine supplements in older adults [331]. Interestingly, whey supplementation produced additional improvements on lean mass and physical function, but only in older adults with frailty or sarcopenia [302]. A systematic review has also shown that citrulline (a non-essential alpha-amino acid) combined with exercise is more effective than exercise or citrulline alone on muscle function and physical performance [332]. Lastly, leucine supplementation combined with exercise requires at least 3 g per day to restore anabolism in older adults [333].

Overall, the level of evidence is, again, poorly reported and a higher number of studies is necessary. Nonetheless, protein or amino acid supplementation might be recommended in close temporal proximity with exercise, but data are controversial [301,334,335]. The available evidence shows no difference in lean mass or muscle strength depending on the protein source (animal vs. vegetal) [336].

In well-nourished subjects, the interactive effect of dietary supplements on muscle parameters is limited and appears to be superior in older adults living with frailty or sarcopenia [302,319]. Supplementation in cases of deficiency still remains valuable, but must not occult the superior effects of resistance exercise training.

### 10.2. Hydroxymethylbutyrate

HMB supplementation combined with resistance exercise in older adults has been shown to improve body composition, compared with exercise alone [337]. Similarly, in older adults undergoing rehabilitation following hip fracture, HMB was associated with functional and muscle mass improvements [338]. The expression of T-cell-specific inflammatory genes was significantly influenced by the combination of exercise and HMB supplementation in older adults with sarcopenia, and this was associated with lower limb muscle strength performance [339]. Conversely, another study showed that HMB only produced negligible benefits, which, as a result, could not be advocated to a larger scale [340].

### 10.3. Vitamin D

In a recent review, no additional effect of vitamin D supplementation and exercise was found for muscle mass and only contradictory results were found in terms of muscle performance [341]. However, in older adults with sarcopenia, the combination of vitamin D supplementation with protein supplementation and exercise has been shown to significantly increase grip strength and has also shown a tendency to increase muscle mass [342].

### 10.4. Polyunsaturated Fatty Acid

Results concerning the synergistic effect of polyunsaturated fatty acid supplementation and exercise on muscle mass and performance are contradictory [306]. Supplementation with omega-3 fatty acids may improve lower body strength and functionality in older adults [343], but has no effect on walking performance [344]. Consequently, there is insufficient evidence to recommend this nutritional intervention for older adults with sarcopenia.

**Key Points**

The combination of exercise and nutritional interventions display limited and controversial results, but it may be an interesting strategy in older adults with sarcopenia and those with deficiencies.

Protein and amino acids supplementation may present a higher interest, especially in older adults with sarcopenia and those with deficiencies regarding their intrinsic effects on muscle and the higher number of studies reported.

## 11. Conclusions

The implementation of a multimodal program including exercise, nutrition and behavioral strategies in the prevention and treatment of frailty in community-dwelling older adults has to be structured around five major steps: (1) screen older adults who are prone to or who may develop frailty with appropriate tools; (2) undertake a comprehensive geriatric assessment including PA level and nutritional assessments; (3) elaborate a structured and progressive multicomponent supervised and group-based exercise program, tailored to individual capacity and objectives; (4) implement the exercise program in feasible, acceptable, accessible and replicable settings; (5) foster older adult’s involvement and empowerment in health-related behaviors (in PA and nutrition) to elicit sustainable changes and maintain benefits. Following this strategy can help to obtain positive outcomes in the short-term, and, above all, in the middle- and long-term. Although epidemiological studies assert that nutrition, among others, underlies frailty and sarcopenia, the adjuvant effects of nutritional interventions to exercise remains limited and controversial. Promoting the whole-diet approach (i.e., emphasizing the overall quality and composition of an individual entire diet) may be an adequate strategy to obtain additional benefits induced by resistance training, before considering supplementation. This narrative review addressed several practical gaps and has proposed evidence-based recommendations to fill them. Trained HCP could use these recommendations to transpose evidence-based research into real-life implementation of prevention programs for older adults with frailty. Regarding exercise training for older adults with frailty, further research is needed (i.e., comparative studies) not only to clarify the optimal dose-response relationship, but also to highlight mechanisms that induce limited exercise responses. Consequently, exercise has to be prescribed according to individual characteristics and needs, in order to enhance exercise responders. Furthermore, stronger evidence is needed to assert that nutritional supplementation is beneficial for older adults with frailty undergoing exercise.

## Figures and Tables

**Table 1:** Composition of a multimodal program including exercise, nutrition and behavioral strategy. ^1^ Supervised Resistance Training; ^2^ Between sets; ^3^ One-Repetition Maximum.^4^ Endurance Training.

	Phase 1 (First 4 Weeks)	Phase 2 (from Week 5 to 8)	Phase 3 (from Week 8 to 12)
**Frequency and time**	2 to 3 days/week of ~1 h each [27,43,50,51,52,53]		
**Supervised duration**	9 to 12 weeks [27,51,53,54,55]		
**Volume in SRT ^1^**	1–3 × (6–10 reps) [50,51,56,57,58]	2–3 × (8–15 reps) [50,51,56,57,58]	2–3 × (10–15 reps) [50,51,56,57,58]
**Rest periods ^2^ in SRT ^1^**	Non-fixed [51,59]	~1 to 2 min [51,59]	~1 to 2 min [51,59]
**Intensity in SRT ^1^**			
**Borg CR10**	2 to 3/10 [27,50,51,60,61]	3 to 5/10 [27,50,51,60,61]	3 to 8/10 [27,50,51,60,61]
**% 1RM ^3^**	20 to 50%	50 to 70%	50 to 85%
**Type of training**	Multicomponent including: Functional-resistance [62,63] Balance [50,64] Endurance between sessions [11,65] Flexibility [43,66]	Multicomponent including: Functional-resistance [21,27,62,63] Balance [50,64] Endurance during and between sessions [11,21,65] Flexibility [43,66]	Multicomponent including: Functional and power-resistance [50,62,63,67] Balance [50,64] Endurance during and between sessions [11,21,65] Flexibility [43,66]
**Exercise selection**	Functional and multi-joint movements with emphasis on the lower limbs [21,68,69]		
**Exercise regimens**	Concentric [50] Isometric [21,70]	Concentric and eccentric [50,71] Isometric [21,70]	High-velocity concentric [67] Eccentric [71] Isometric [21,70]
**Volume in ET ^4^**	Reducing time spent in sedentary behaviors [10,11]	120 to 150 min/week of light-to-moderate PA [72]	150 to 300 min/week of light-to-moderate PA [12]
**Intervention settings**	Unsupervised home-based for robust older adults with pre-frailty [20,73] Supervised group-based for older adults with frailty [20,74] Individual interventions for older adults with severe limitations and frailty [65,75] Preferably, use free weight and/or small equipment [62,68]		
**Behavioral strategy**	Knowledge acquisition [76,77,78] Goals settings Self-efficacy Intrinsic motivation Accessible support and follow-up		
**Nutrition**	Mainly caloric and protein intake [79,80,81,82] Protein sources and quality		

**Table 2:** Summary table of each section’s key points.

Screening strategies	Screening strategy has to be based on performant and feasible tools designed for the intended setting. Physical performances could be appropriate surrogates of frailty indexes and can be easily assessed. PA is a predominant risk factor of frailty and must be a primary variable to assess. Self-screening using new technologies represents a great opportunity to move forward in this field and can be used with confidence.
Assessment methodologies	Assessments of physical capacity, nutritional intake and physical activity level as part of a CGA must be undertaken to deliver personalized interventions. A complete assessment ensures the safety, the acceptability and the efficacy of the exercise program. Assessments should be followed by building an action plan to involve and empower older adults with frailty during the multimodal program. Action plans should be focused on personalized goal setting and provide knowledge acquisition in exercise and nutrition.
Exercise training components	Conception, elaboration and implementation of a tailored multicomponent exercise program for older adults with frailty have to integrate appropriate frequency, intensity, type, time, volume and progressiveness to warrant individual benefits. Program has to be structured around 2 to 3 non-consecutive sessions per week of ~1 h each, at a progressive low-to-moderate-to-vigorous intensity of 20 to 85% 1RM or RPE 2 to 8/10, for a minimum of 12 weeks, with an emphasis on functional resistance training (strength and power) and endurance. Progressive volume has to range from 1 to 4 sets of 6 to 15 repetitions, with a rest period of 1 to 2 min depending on exercise tolerance and chronic conditions.
Exercise program contents	Exercise selection has to emphasize lower limb exercises, in particular those recruiting femoral muscles, complemented by additional exercises of the upper body (especially grip and trunk). Priority should be given to functional multi-joint exercises to improve physical function. For older adults with frailty, functional multi-joint exercises at bodyweight should be included at first before progressing with additional resistance. Overall, concentric contractions should be predominant over eccentric, but progressing to eccentric contractions should be considered as a safe and relevant method to improve training performance and physical function.
Intervention settings	Exercise intervention settings have to be in line with feasibility, acceptability, accessibility and replicability. Resistance training based on free-weight and small equipment is more advisable. For older adults with frailty and/or chronic health conditions, supervised group-based interventions are recommended as they provide several advantages that can foster involvement, empowerment and self-efficacy. Unsupervised or remote home-based exercise programs are safe and effective but should be more indicated to robust older adults or those with pre-frailty.
Behavioral strategies	Behavioral strategies are strongly advocated to elicit sustainable changes in PA and prevent detraining effects. Behavioral strategies should include specific goal-setting behavior, self-efficacy, intrinsic motivation for PA, outcome expectancies and continued peer-social support.
Nutrition	Balanced diet covering caloric and protein demands that also provide essential nutrients is crucial to prevent sarcopenia and frailty. Vitamin D, omega-3 fatty acids and antioxidants should have special considerations in the diet. Evidence for nutritional interventions alone to improve sarcopenia outcomes does not support their implementation.
Exercise with nutritional interventions	The combination of exercise and nutritional interventions displays limited and controversial results but it may be an interesting strategy in older adults with sarcopenia and those with deficiencies. Protein and amino acids supplementation may present a higher interest, especially in older adults with sarcopenia and those with deficiencies regarding their intrinsic effects on muscle and the higher number of studies reported.

## Data Availability

No new data were created or analyzed in this study. Data sharing is not applicable to this article.
